# Nanoparticle‐Based Tolerogenic Vaccines: Next‐Generation Strategies for Autoimmune and Allergic Disease Therapies

**DOI:** 10.1002/anie.202524097

**Published:** 2025-12-26

**Authors:** Benjamin E. Nachod, Ajay S. Thatte, Rohan Palanki, Michael J. Mitchell

**Affiliations:** ^1^ Department of Bioengineering University of Pennsylvania Philadelphia Pennsylvania 19104 USA; ^2^ Penn Institute for RNA Innovation Perelman School of Medicine University of Pennsylvania Philadelphia Pennsylvania 19104 USA; ^3^ Institute for Immunology Perelman School of Medicine University of Pennsylvania Philadelphia Pennsylvania 19104 USA; ^4^ Cardiovascular Institute Perelman School of Medicine University of Pennsylvania Philadelphia Pennsylvania 19104 USA; ^5^ Institute for Regenerative Medicine Perelman School of Medicine University of Pennsylvania Philadelphia Pennsylvania 19104 USA; ^6^ Abramson Cancer Center Perelman School of Medicine University of Pennsylvania Philadelphia Pennsylvania 19104 USA

**Keywords:** Autoimmunity, Allergy, Immunomodulatory materials, Immune tolerance, Nanoparticle design strategies, Vaccine

## Abstract

The concurrent rise of autoimmune diseases, which affect nearly 10% of the global population, along with allergic conditions such as asthma, food allergy, and atopic disease, together pose substantial health and economic burdens. Traditional therapies rely on systemic immunosuppression that temporarily mitigates symptoms but often compromises protective immunity, increases infection and malignancy risk, and fails to restore central or peripheral tolerance. These limitations underscore the need for antigen‐specific strategies capable of re‐establishing durable immune balance. Tolerogenic vaccines have emerged as a promising solution by retraining the immune system to restore antigen‐specific tolerance while preserving normal host defense, though challenges remain in efficiently targeting antigen‐presenting cells (APCs), avoiding their overactivation, and minimizing off‐target effects. Nanoparticles provide a versatile platform to address these hurdles, as their size, composition, and surface modifications can be tailored to direct biodistribution, enhance antigen delivery, and modulate immune signaling. By co‐delivering antigens and immunomodulators in programmable ways, nanoparticles offer a pathway to overcome key translational barriers and achieve precise immune reprogramming. This review explores how advances in nanomedicine are being applied to tolerogenic vaccines, focusing on three areas: (1) current nanoparticle platforms, (2) the role of biomaterial selection, and (3) multifunctional engineering strategies, while also considering the clinical outlook and translational challenges of bringing these therapies from bench to bedside.

## Graphical abstract

Nanoparticle‐based tolerogenic vaccines harness controlled antigen delivery and immunomodulation to establish tolerance in autoimmunity and allergy. This review outlines how nanoparticle design (size, shape, composition, administration route) influences biodistribution and immune‐cell targeting. We highlight preclinical and clinical progress, translational challenges, and future directions including biomaterial innovation and tolerance‐guiding adjuvants.

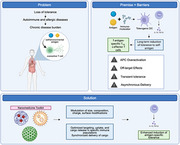

## 1 Introduction

Once considered rare, autoimmune diseases now affect approximately 10% of people worldwide, with the prevalence of these conditions increasing by 42% between 2000 and 2019.^[^ ^1^ ^]^ Autoimmune diseases are characterized by a breakdown of immunological tolerance, resulting in pathological immune responses against self‐antigens. Depending on the specific disorder, tissue damage may be mediated by autoreactive CD4⁺ and CD8⁺ T cells, inflammatory antigen‐presenting cells (APCs), immune complexes, or autoantibody‐producing B cells. Examples range from systemic pathologies such as rheumatoid arthritis (RA) and systemic lupus erythematosus (SLE), to tissue‐specific conditions including type 1 diabetes (T1D), Graves’ disease, and multiple sclerosis (MS). Allergic diseases, by contrast, arise from inappropriate activation of immune pathways normally dedicated to parasitic defense against innocuous environmental antigens (allergens). These responses are typically driven by allergen‐specific IgE, Th2 cells, mast cells and basophils, and the prevalence now impacts 10–30% of the global population.

Autoimmune and allergic diseases affect individuals across all demographics; however, they are particularly prevalent in women and children.^[^ ^2^, ^3^ ^]^ In addition, their burden is more pronounced in industrialized nations, where changing environmental exposures and hygienic living conditions are hypothesized to skew immune development.^[^ ^4^ ^]^ The economic implications of these conditions are significant. Autoimmune diseases account for over $100 billion per annum in direct and indirect healthcare costs in the United States,^[^ ^5^ ^]^ while allergic diseases result in billions of dollars more in emergency care, lost productivity, and long‐term pharmaceutical reliance.^[^ ^6^ ^]^

Current management strategies for autoimmune and allergic diseases rely on systemic immunosuppression. While effective at symptom mitigation, these therapies can heighten the risk of infection and malignancy, delay wound healing, and adversely affect the kidney, liver, and bone.^[^ ^7^, ^8^ ^]^ Thus, there is a critical need for therapies capable of inducing long‐term and specific immune tolerance to disease‐causing antigens while preserving normal host defense.

Tolerogenic vaccines are a novel therapeutic class that can potentially address this challenge. Unlike traditional vaccines that activate the immune system for disease prevention, tolerogenic vaccines suppress the immune response to specific antigens for disease resolution.^[^ ^9^, ^10^ ^]^ To induce antigen‐specific tolerance, tolerogenic signals must be delivered in a precise and controlled manner. Emerging approaches use nanoparticles as delivery vehicles to present tolerogenic cues to the cells and tissues responsible for tolerance induction. Because autoimmune and allergic diseases are driven by distinct effector mechanisms, the design of tolerogenic nanoparticle strategies must be tailored to the underlying immunopathology. In allergic disease, pathology is dominated by Th2‐skewed immunity and IgE‐mediated activation of mast cells and basophils; thus, effective interventions often aim to suppress IgE class switching, reprogram Th2 cells, or induce allergen‐specific IL‐10–producing regulatory T cells (T_regs_).^[^ ^11^, ^12^ ^]^ In contrast, many autoimmune diseases (such as MS, RA, and T1D) are driven by autoreactive CD4⁺ and CD8⁺ T cells, inflammatory APCs, and pathogenic autoantibody production.^[^ ^13^, ^14^, ^15^ ^]^ In such cases, nanoparticle design may prioritize targeting dendritic cells, macrophages, or autoreactive antigen‐specific T cell and B cell subsets to induce deletion, anergy, or regulatory differentiation. Consequently, optimal antigen format, delivery route, and immunomodulatory cues differ across disease classes, and disease‐specific mechanistic understanding must guide nanoparticle engineering for antigen‐specific tolerance. In this review, we examine the key design principles of nanoparticles used in tolerogenic vaccines and highlight next‐generation engineering strategies aimed at realizing their potential in treating autoimmune and allergic disease.

## 2 Tolerogenic Vaccines: Development and Mechanisms of Action

Traditional vaccines have three major components: 1) an antigen that triggers immunity, 2) an adjuvant that enhances immune activation, and 3) a delivery system that ensures antigen reaches the appropriate immune cells. Antigen‐adjuvant pairs activate pattern‐recognition receptors on dendritic cells (DCs), leading to upregulation of CD80/86 on the cell surface, secretion of IL‐12, and priming of naïve lymphocytes.^[^ ^16^, ^17^, ^18^, ^19^ ^]^ The resultant robust B cell mediated antibody response and potent effector/memory T cell proliferation provide durable protection against target pathogens.^[^ ^20^, ^21^ ^]^ In autoimmune and allergic disease, the traditional vaccine paradigm is unsuitable, since the immune system is already inappropriately primed against self‐antigens or environmental antigens. Instead, the desired outcome for vaccination against these conditions is to convey immune tolerance, a state in which the immune system does not respond to specific antigens.

In normal physiology, there are two mechanisms of immune tolerance. Central tolerance takes place in primary lymphoid organs, whereby self‐reactive B cells and T cells are eliminated in the bone marrow and thymus, respectively.^[^ ^22^, ^23^ ^]^ Peripheral tolerance acts as a fail‐safe mechanism for self‐reactive lymphocytes that have escaped central tolerance, whereby T_regs_, inhibitory ligands, and anti‐inflammatory cytokines in the circulation and peripheral tissues suppress autoreactive or allergen‐specific lymphocytes.^[^ ^24^, ^25^ ^]^ In autoimmune and allergic diseases, breakdown of these mechanisms underlies chronic pathology.^[^ ^26^, ^27^, ^28^, ^29^, ^30^ ^]^

Tolerogenic vaccines invert the logic of traditional vaccination. By presenting disease‐relevant antigens in the presence of active immunomodulators (tolerogenic cues), tolerogenic vaccines push DCs to downregulate the costimulatory molecules CD80/86, increase expression of inhibitory ligands (e.g., PD‐L1, ICOSL), and secrete anti‐inflammatory cytokines such as IL‐10 and TGF‐β.^[^ ^31^ ^]^ This leads to the generation and expansion of antigen‐specific Foxp3^+^ T_regs_, including thymic‐derived T_regs_ (tT_regs_), peripherally induced T_regs_ (pT_regs_), and type 1 regulatory (Tr1) cells, that reinforce peripheral tolerance and actively suppress autoreactive and allergen‐specific T cells through contact‐dependent and cytokine‐mediated mechanisms.^[^ ^32^, ^33^ ^]^ There are several distinct formats for tolerogenic vaccines. Peptide‐based vaccines deliver short self‐epitopes to induce reactive T cell anergy or deletion.^[^ ^34^, ^35^, ^36^ ^]^ Cell‐based vaccines leverage ex vivo‐generated tolerogenic dendritic cells (tolDCs) or T_regs_ to modulate immunity after reinfusion.^[^ ^37^ ^]^ DNA‐based and mRNA‐based platforms encode autoantigens or tolerogenic proteins for in situ immunomodulation.^[^ ^38^ ^]^

Despite their conceptual elegance, tolerogenic vaccines face several practical barriers: (i) antigens and tolerogenic cues are conveyed via fragile cargos such as peptides and mRNA that require protection from rapid degradation; (ii) antigens and tolerogenic cues must be delivered efficiently to the appropriate APC subset (e.g., lymph node–resident DCs, liver sinusoidal endothelial cells) to ensure stable induction of a tolerogenic phenotype; (iii) antigens and tolerogenic cues must be delivered synchronously to the same APC to achieve antigen‐specific immune reprogramming. Nanoscale materials such as lipid or polymer nanoparticles, nanoconjugates, and stimuli‐responsive nanogels offer a versatile toolkit to overcome these challenges. By tuning size, surface chemistry, pharmacokinetics, and stimulus‐responsiveness, these delivery platforms can shield antigens, co‐encapsulate immunomodulators at defined stoichiometries, traffic efficiently through lymphatics, achieve cell‐specific uptake, and release cargos in a controlled fashion within target endosomal or cytosolic compartments. Thus, the design and engineering of nanoparticle platforms is critical for tolerogenic vaccine development.

## 3 Role of Nanoparticle Selection in Tolerogenic Vaccine Development

Nanoparticles are a clinically advanced delivery platform for vaccine development, boasting strong biocompatibility and delivery profiles. The modularity of nanoparticles enables intentional design for vaccine applications. For tolerogenic vaccines, nanoparticles must deliver diverse nucleic acid, peptide, or small molecule cargos to target immune cells and induce antigen‐specific tolerance without systemic immune activation. Biomaterial selection, nanoparticle physicochemical properties, and route of vaccine administration shape the safety and efficacy of tolerogenic vaccines.

### 3.1 Influence of Nanoparticle Composition

**Figure 1:**
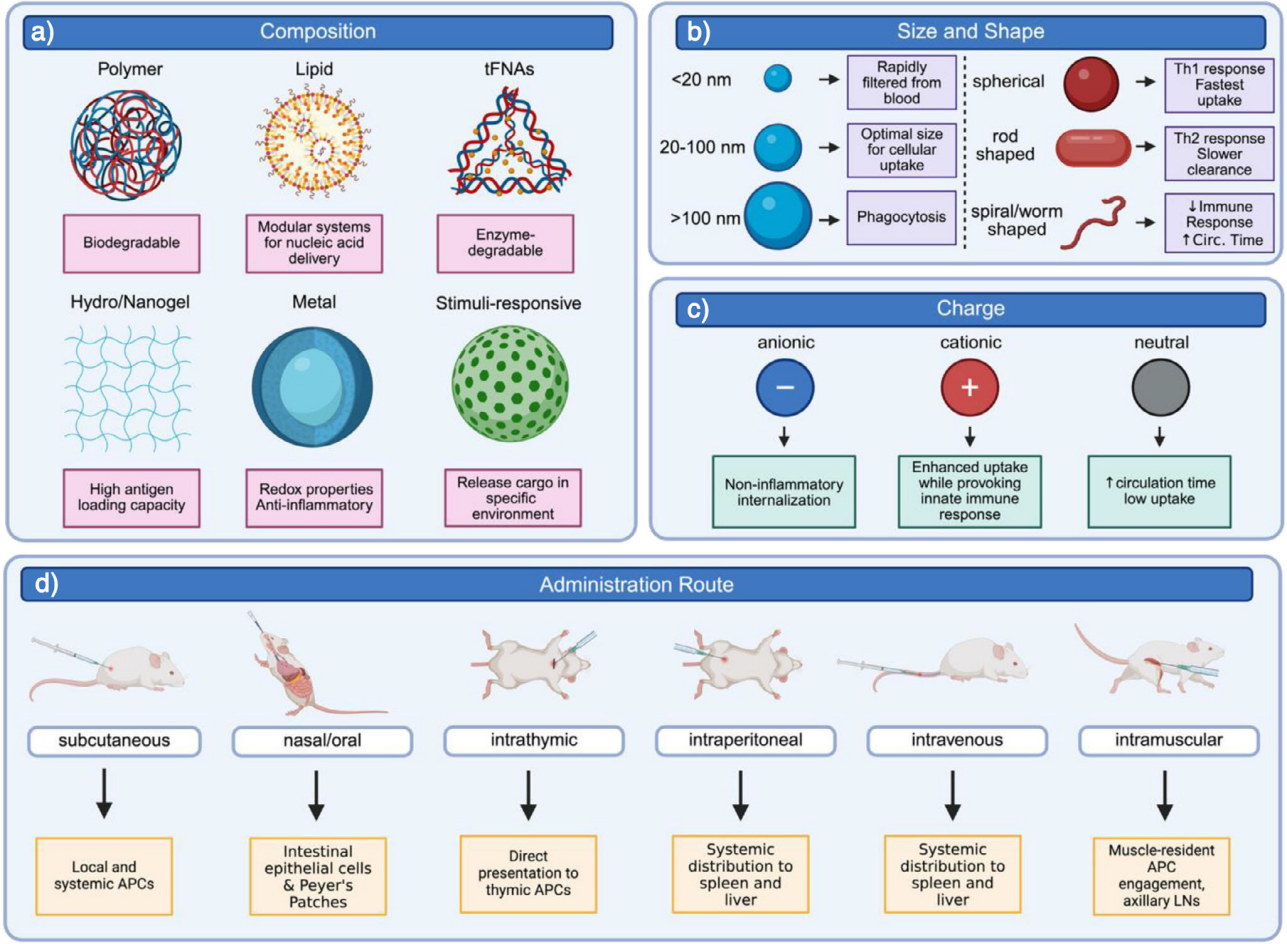
The immunological performance of tolerogenic nanoparticles is dictated by material composition, physicochemical properties, and route of administration. a) Composition. Polymers, lipids, tetrahedral framework nuceic acids (tFNAs), hydrogels, metals, and stimuli‐responsive carriers offer distinct benefits such as biodegradability, nucleic acid delivery, redox activity, or environment‐specific release. b) Size and shape. Optimal cellular uptake occurs at 20–100 nm; spherical particles bias toward Th1 responses, whereas rod or worm‐like particles show slower clearance and different immune polarization. c) Charge. Cationic carriers enhance cellular uptake but risk inflammation, anionic particles support non‐inflammatory internalization, and neutral formulations extend circulation with lower uptake. d) Administration route. Delivery pathway directs biodistribution: subcutaneous administration reaches systemic APCs, nasal/oral administration promotes mucosal tolerance, intrathymic administration targets thymic APCs, intraperitoneal and intravenous administration results in systemic biodistribution primarily in the liver, spleen, and circulating APCs, and intramuscular administration engages local APCs and draining lymph nodes. Together, these parameters form a design toolkit for tuning tolerogenic responses.

The chemical composition of nanoparticles dictates their biodistribution, degradation kinetics, and immune interactions in vivo (Figure 1a). For example, biodegradable poly(lactic‐co‐glycolic) acid (PLGA) polymers accumulate in immune‐tolerant organs like the liver and spleen. Within the liver, the principal tolerogenic APCs include Kupffer cells and liver sinusoidal endothelial cells (LSECs), both of which constitutively express inhibitory cytokines and co‐inhibitory molecules that favor antigen‐specific tolerance. In the spleen, tolerogenic antigen handling is mediated by splenic dendritic cells, marginal zone macrophages, and regulatory B cell subsets, which integrate peripheral antigen signals to support T_reg_ induction.^[^ ^35^, ^39^, ^40^, ^41^, ^42^, ^43^, ^44^, ^45^, ^46^ ^]^ Nanoparticle formulations that preferentially traffic to these populations can therefore leverage their intrinsic immunoregulatory properties to promote systemic tolerance. Consistent with this concept, anionic 1,2‐Distearoyl‐sn‐glycero‐3‐phospholycerol (DSPG) liposomes have been shown to expand antigen‐specific T_reg_ and ameliorate disease in murine models of atherosclerosis, illustrating how lipid composition can directly bias nanoparticle formulations toward tolerance.^[^ ^34^ ^]^ The biodistribution, endosomal escape efficiency, and innate immunogenicity of lipid nanoparticles (LNPs) can be fine‐tuned to promote nucleic acid delivery to tolerogenic cell types by substituting ionizable lipids, adjusting molar ratios of helper lipids (cholesterol, DSPC, PEG‐lipids), or varying the chemical structure of lipid tails.^[^ ^47^, ^48^, ^49^, ^50^ ^]^ Stimuli‐responsive carriers, such as ethoxy acetalated dextran (Ace‐DEX) nanoparticles, hydrolyze under acidic conditions, enabling precise endosomal antigen release.^[^ ^51^ ^]^ Antigen released within the endosome is subject to proteolysis in the MHC class II compartment and subsequently loaded onto MHC class II molecules for presentation to CD4⁺ T helper cells. If the release mechanism allows cytosolic escape, antigen can enter the cross‐presentation pathway and be delivered to the proteasome, enabling loading onto MHC class I molecules and presentation to CD8⁺ T cells, a pathway which depending on the activation context may lead to either immune tolerance through deletion or anergy of autoreactive T cells or immune activation by priming CD8⁺ effector T cells. Redox‐responsive polydopamine (PDA) particles release their payload in glutathione‐rich intracellular environments, synchronizing intracellular processing with tolerogenic signaling.^[^ ^52^, ^53^ ^]^ Metal‐based carriers, such as gold and cerium oxide nanoparticles, possess intrinsic redox properties that can contribute to anti‐inflammatory immune modulation.^[^ ^54^, ^55^ ^]^ Nanogels and tetrahedral framework nucleic acids (tFNAs) provide enzyme‐degradable scaffolds with high antigen‐loading capacity and low immunogenicity.^[^ ^56^, ^57^, ^58^, ^59^ ^]^

### 3.2 Physicochemical Properties: Size, Shape, and Charge

Nanoparticle uptake is dependent on physicochemical properties, including size, shape, and charge (Figure 1b,c). Nanoparticles under 100 nm can enter lymphatic vessels and access lymph nodes, the central sites for tolerogenic APC engagement.^[^ ^60^ ^]^ Gold nanoparticles synthesized at precise diameters showed size‐dependent uptake, antigen‐specific T cell priming, and lymph node retention.^[^ ^61^ ^]^ Similarly, nanoparticle size modulates retention in lymphoid follicles and antigen presentation efficiency.^[^ ^54^, ^62^ ^]^ Nanoparticle shape has been shown to influence immune polarization. For example, polystyrene particles loaded with full ovalbumin (OVA) protein induced distinct T cell responses: small spherical particles biased toward Th1, while long rod‐shaped ones favored Th2.^[^ ^63^ ^]^ Koirala et al. showed that rod‐, sphere‐, and worm‐shaped nanoparticles elicited different humoral and mucosal immunity profiles when delivered via oral or nasal routes.^[^ ^64^ ^]^ Moreover, this shape‐effect appears consistent across different nanoparticle materials. For example, glyco‐nanoparticles and peptide‐based fiber/rod nanostructures also display shape‐dependent variations in antigen uptake, dendritic‐cell activation and downstream Th‐cell skewing.^[^ ^65^, ^66^ ^]^ Mechanistically, this is thought to arise because spherical particles are internalized more rapidly by dendritic cells and favor endosomal escape and cross‐presentation via MHC I, thereby promoting Th1 responses, whereas rod‐shaped particles exhibit slower uptake, remain predominantly in endo‐lysosomal compartments and favor MHC II‐dependent loading and Th2 responses.^[^ ^67^ ^]^ Finally, nanoparticle electrostatic charge influences both cell membrane interactions and intracellular endosomal escape. Cationic particles may enhance cell uptake but frequently provoke innate immune activation, whereas anionic particles containing PS or DSPG are preferentially internalized in a non‐inflammatory manner that supports tolerogenic programming.^[^ ^68^, ^69^ ^]^

### 3.3 Nanoparticle Administration Routes in Tolerogenic Vaccination

The route of nanoparticle administration dramatically affects antigen processing and resultant systemic or local immune response (Figure 1d). Depending on nanoparticle design and disease context, each route of administration may promote distinct tolerogenic outcomes via exposure to specific APC populations and immune organs (Table 1).^[^ ^70^ ^]^ Oral delivery leverages gut‐associated lymphoid tissue (GALT) and the inherently tolerogenic mucosal environment, enabling induction of T_regs_ and allergen‐specific hypo‐responsiveness. However, it faces challenges of enzymatic degradation, variable absorption, and high antigen dose requirements.^[^ ^44^, ^71^, ^72^ ^]^ Intranasal administration accesses the nasal‐associated lymphoid tissue (NALT)—a lymphatic‐rich mucosal site capable of inducing mucosal, humoral and cellular immunity. However, deposition variability, low antigen availability and weak immunogenicity remain major barriers.^[^ ^64^, ^73^, ^74^ ^]^ Subcutaneous/intradermal delivery offers precise dosing and efficient access to skin‐resident APCs, yet the skin lacks the default tolerogenic bias of mucosal sites and may strongly engage innate immune pathways.^[^ ^40^, ^75^ ^]^ Intravenous and intraperitoneal administration affords reproducible systemic biodistribution and access to central tolerogenic organs such as liver and spleen, but risks broad uptake by the mononuclear phagocyte system and less specificity for lymphoid‐tissue targeting.^[^ ^76^, ^77^ ^]^

**Table 1:** Summary of nanoparticle administration routes and associated immune targeting outcomes. Different administration routes influence nanoparticle trafficking, antigen processing, and engagement with distinct APC populations and organs.

Administration route	Description of immune targeting
Subcutaneous (s.c.)	Used widely for PLGA particles, targets local and systemic APCs.^[^ ^40^, ^75^ ^]^
Nasal and Oral	Favor mucosal tolerance and antigen uptake via intestinal epithelial cells and Peyer's patches.^[^ ^44^, ^64^, ^72^ ^]^
Intrathymic (i.t.)	Directly presents antigen to thymic APCs, promoting central deletion or T_reg_ induction.^[^ ^78^ ^]^
Intraperitoneal (i.p.)	Bypasses epithelial barriers, often used in preclinical models.
Intravenous (i.v.)	Enables systemic distribution, particularly to the spleen and liver.^[^ ^76^ ^]^
Intramuscular (i.m.)	Common for clinical vaccines; depot effect with muscle‐resident APC engagement. Activated APCs travel to axillary draining lymph node.^[^ ^79^ ^]^

When the goal is systemic immune tolerance, intravenous delivery has distinct advantages: antigens or nanoparticles delivered through the bloodstream reach the spleen and liver, organs rich in tolerogenic APCs including marginal‐zone macrophages, LSECs, and Kupffer cells. These cells naturally promote tolerogenic outcomes (IL‐10/TGF‐β production, T cell anergy, and Foxp3⁺ T_reg_ induction) making them well‐suited for systemic tolerance.^[^ ^80^ ^]^ Tuning particle size, surface chemistry, and dose allows systemic delivery to engage broad immune compartments instead of staying confined to one site. However, systemic delivery is not the optimal administration route for all disease applications. In diseases driven by tissue‐resident immunity or mucosal antigen exposure, such as food allergy or gut autoimmune conditions, mucosal or intradermal/subcutaneous routes may better target relevant immune niches. The choice of route of administration requires consideration of the site of antigen presentation, whether circulating or tissue‐resident immune compartments need modulation, and the potential for off‐target effects from systemic distribution.

Taken together, intrinsic nanoparticle properties and their routes of administration comprise a toolkit for engineering tolerogenic vaccines. By tuning physicochemical properties, nanoparticles can be programmed to accumulate in organs such as the liver,^[^ ^42^, ^43^, ^47^ ^]^ lymph nodes,^[^ ^53^, ^61^, ^81^ ^]^ or spleen^[^ ^48^ ^]^ and target key immune cell subsets such as dendritic cells,^[^ ^82^, ^83^ ^]^ B cells,^[^ ^45^, ^84^, ^85^ ^]^ and T cells. In the context of antigen‐specific tolerance, nanoparticle platforms exploit this biodistribution and immune‐cell targeting paradigm to selectively engage specific immune cell populations or immunoregulatory pathways. DCs process and present antigen via MHC class I and II pathways, driving T_reg_ responses or deleting antigen‐specific helper T cells. B cells can present antigen or be targeted directly to deplete pathogenic clones and induce IL‐10‐producing regulatory B cells. In the case of direct targeting, instead of relying on antigen presentation, the purpose is to modulate the functional state of T cells. This includes expanding naïve CD4⁺ T cells into Foxp3⁺ T_regs_, silencing CD8⁺ autoreactive populations through apoptosis or anergy, or stabilizing existing T_reg_ subsets via receptor agonists or cytokine responses. The choice of T cell subset that is targeted influences antigen format, choice of co‐delivered immunomodulators, and delivery route. Reduction in immunogenicity or cytotoxicity can be achieved through surface modifications that impact nanoparticle physicochemical properties.^[^ ^69^, ^75^ ^]^ Biodistribution and antigen processing by distinct APC populations are dependent on route of nanoparticle administration. These design variables allow for control over antigen and immunomodulator delivery, which in turn enables selective engagement of tolerance‐inducing pathways. Thus, the design and engineering of nanoparticle platforms are critical for tuning the immune response of tolerogenic vaccines.

## 4 Strategies for Engineering Nanoparticle‐Based Tolerogenic Vaccines

Designing nanoparticle‐based tolerogenic vaccines requires targeting specific immune cell populations, enhancing intracellular antigen delivery, and skewing immune responses toward tolerance.^[^ ^86^ ^]^ To achieve these key outcomes, several classes of nanoparticle platforms have been designed that combine cell targeting, tolerogenic signaling, and antigen presentation (Table 2).

### 4.1 Bioconjugation and Chemistry‐Driven Strategies

Bioconjugation strategies utilize chemical modification to enhance nanoparticle function, stability, and immune specificity. Three strategies for bioconjugation include the direct use of bioconjugates, polymer modification during nanoparticle formulation, or polymer modification after nanoparticle formulation. The porosity of nanoparticles influences which of these techniques can be used. Porous nanoparticles allow for passive antigen loading by diffusion, whereas nonporous platforms require covalent attachment of cargo via techniques like maleimide‐thiol or EDC/NHS coupling.^[^ ^87^ ^]^

**Figure 2:**
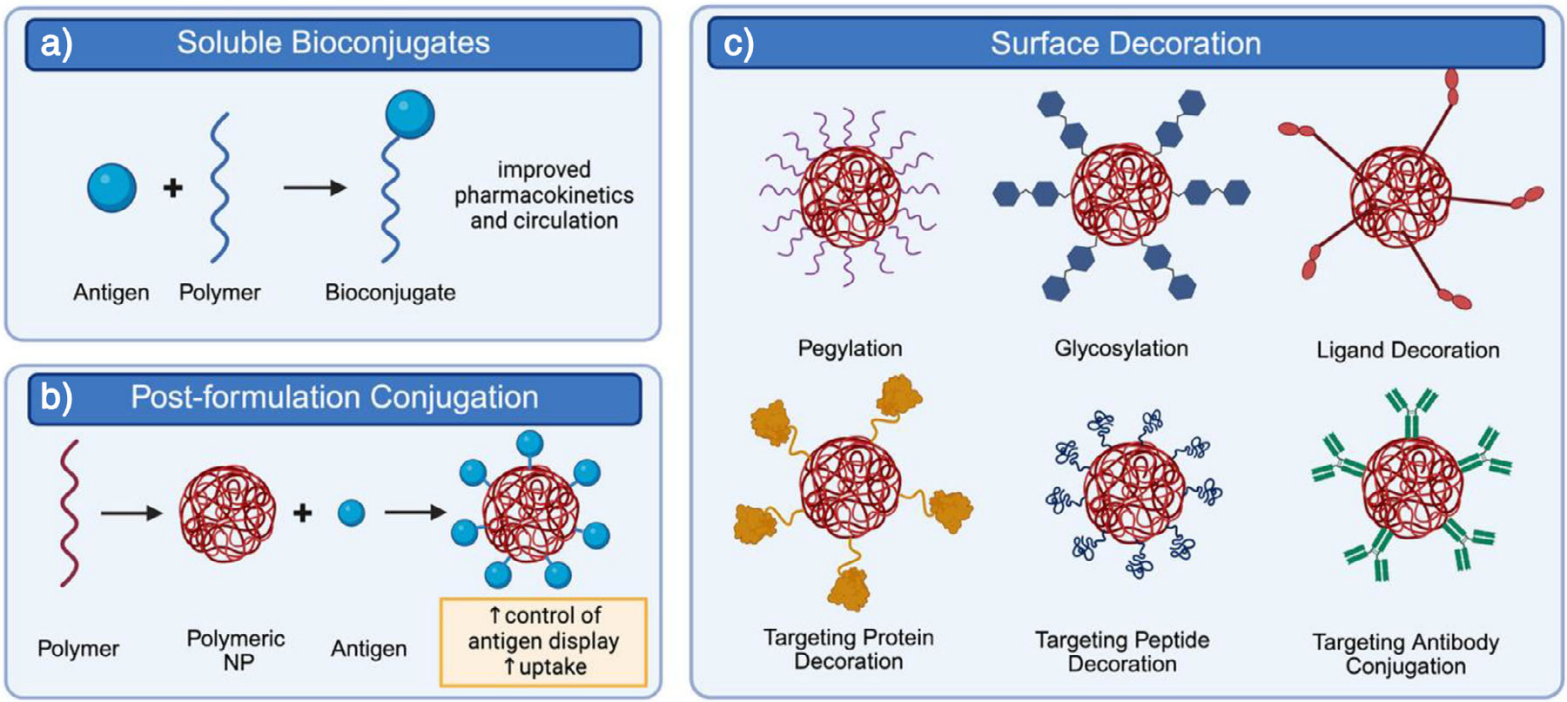
Chemical conjugation offers versatile ways to modify antigen delivery systems and modulate immune responses. a) Soluble bioconjugates. Direct conjugation of antigens to soluble polymers, such as PEG, improves pharmacokinetics, extends circulation time, and enhances antigen stability without requiring particulate carriers. b) Post‐formulation conjugation. Antigens can also be tethered to pre‐formed nanoparticles, allowing greater control over antigen density and orientation on the surface. c) Surface decoration. Beyond simple antigen tethering, nanoparticles can be decorated with diverse moieties to improve targeting and modulate immune interactions. Examples include PEGylation to reduce nonspecific uptake, glycosylation to engage lectin receptors and direct trafficking to tolerogenic APCs, and ligand conjugation for organ‐ or cell‐specific delivery. Surface decoration with proteins, peptides, or antibodies enables precise targeting to immunologically relevant receptors, further enhancing tolerogenic outcomes. Collectively, these approaches illustrate how bioconjugation and surface engineering expand the functional versatility of nanoparticles, enabling both improved pharmacological performance and immune‐specific programming.

Soluble bioconjugates, such as PEG‐antigen complexes, improve antigen pharmacokinetics and immune processing without forming particulate carriers (Figure 2a). For example, Pfeil et al. synthesized OVA_323–339_ peptides conjugated to PEG of various molecular weights using maleimide‐thiol chemistry and demonstrated enhanced peptide half‐life and increased antigen‐specific splenic Foxp3^+^ T cell frequencies in DO11.10 mice after subcutaneous injection.^[^ ^88^ ^]^ This PEGylation method was further applied to MOG_35–55_ peptides in a murine EAE model, whereby prophylactic administration of the PEG‐antigen platform prevented disease progression, although therapeutic use after disease onset was ineffective. In the design of PEG‐antigen peptide conjugates, the peptide motif primarily determines which immune cell subsets or lymphoid tissues are engaged (for example, via DC‐receptor binding or B‐cell receptor recognition), whereas PEGylation serves mainly to extend circulation half‐life, reduce proteolysis and immunogenicity, and promote lymphatic drainage from the injection site, with studies showing enhanced access to draining lymph nodes.^[^ ^89^ ^]^ Thus, PEGylation simultaneously serves to extend circulation time and amplify tolerogenic signals when conjugated to antigen, coupling delivery and immunomodulation in a single system.

Post‐formulation bioconjugation, such as antigen tethering to nanoparticle surfaces, enables external antigen display (Figure 2b). Phan et al. conjugated MOG_35–55_ to lignin NP surfaces using EDC/NHS chemistry, enhancing antigen density and promoting uptake by dendritic cells.^[^ ^90^ ^]^ Lignin is a naturally occurring polyphenolic polymer whose abundant phenolic hydroxyl and methoxyl groups provide strong radical‐scavenging, antioxidant activity, and amenability to functionalization make it a promising material for nanoparticle fabrication and controlled antigen delivery.^[^ ^91^ ^]^ Lignin's highly cross‐linked aromatic polymer backbone offers numerous reactive sites for chemical conjugation (e.g., amination, acylation, sulfonation), thereby enabling versatile modification of its surface chemistry, solubility, and interaction with biological systems. In EAE mice, MOG‐lignin NPs reduced CD86 and MHC‐II expression on DCs, decreased splenic oxidative stress, and increased Foxp3^+^ T_reg_ proportions, resulting in complete disease suppression. Smarr et al. similarly conjugated soluble antigen to the outer surface of PLG NPs, which outperformed antigen‐PLG conjugates in Th2 inhibition and prevention of airway eosinophilia.^[^ ^92^ ^]^

Nanoparticles can be further engineered by decorating their surfaces with glycan ligands or targeting peptides to direct trafficking to immune‐tolerant organs like the liver (Figure 2c). Wen et al. and Qiao et al. coated PLGA NPs with mannan to target the CD206 mannose receptor on DCs and macrophages.^[^ ^82^, ^93^ ^]^ In models of allergy and RA, mannan‐modified NPs enhanced DC uptake, induced tolerogenic profiles, and reduced disease‐associated inflammation. Similarly, Liu et al. used ApoB‐conjugated PLGA NPs to direct antigen delivery to liver sinusoidal endothelial cells (LSECs), which promote antigen‐specific T_reg_ expansion.^[^ ^43^ ^]^ ApoB‐decorated particles significantly reduced allergen‐specific IgE levels and Th2 inflammation in models of peanut anaphylaxis and pulmonary sensitization.

Nanoparticles can also be modified to promote interaction or evasion with specific immune cell populations. For example, Lewis et al. targeted DC integrins by conjugating RGD and P‐D2 peptides to NP surfaces.^[^ ^94^ ^]^ Peptide‐conjugated nanoparticles enhanced DC association without activating pro‐inflammatory pathways, highlighting their potential for directing antigens to tolerogenic DC subsets. Li et al. addressed the potential immunogenicity of NPs by PEGylating the surface of PLGA nanoparticles encapsulating OVA to reduce non‐specific immune cell uptake.^[^ ^75^ ^]^ PEGylated PLGA NPs improved lymphoid tissue accumulation and induced antigen‐specific tolerance without systemic inflammatory response, likely due to preferential engagement with non‐inflammatory APCs.

Bioconjugation strategies may be combined for a single nanoparticle platform. Srivastava et al. designed PEGylated liposomes bearing both OVA antigen and CD22L for B cell targeting.^[^ ^84^ ^]^ In a K/BxN mouse model of RA, dual‐display liposomes suppressed autoantibody titers from B cells and pro‐inflammatory responses from T cells, while single‐ligand or antigen‐only liposomes failed to elicit this response. Brzezicka et al. developed a similar platform, whereby hybrid nanoparticles that co‐presented antigens and the ligand CD22L induced T and B cell‐mediated tolerance in autoimmune arthritis.^[^ ^45^ ^]^ Thus, combinatorial bioconjugation of nanoparticles facilitates the modular assembly of synergistic signals for tolerogenic vaccines.

### 4.2 Synchronized Delivery of Antigen and Immunomodulatory Cargos

Effective induction of antigen‐specific tolerance requires modulation of multiple components of the immune system simultaneously. Nanoparticles enable precise co‐delivery of antigens and immunomodulators, balancing immune activation and suppression in both innate and adaptive responses. A consistent theme across studies is that synchronized delivery, whether through co‐encapsulation in a single particle or co‐administration of complementary nanoparticles, produces stronger and more durable tolerogenic effects than delivering cargos separately.

**Figure 3:**
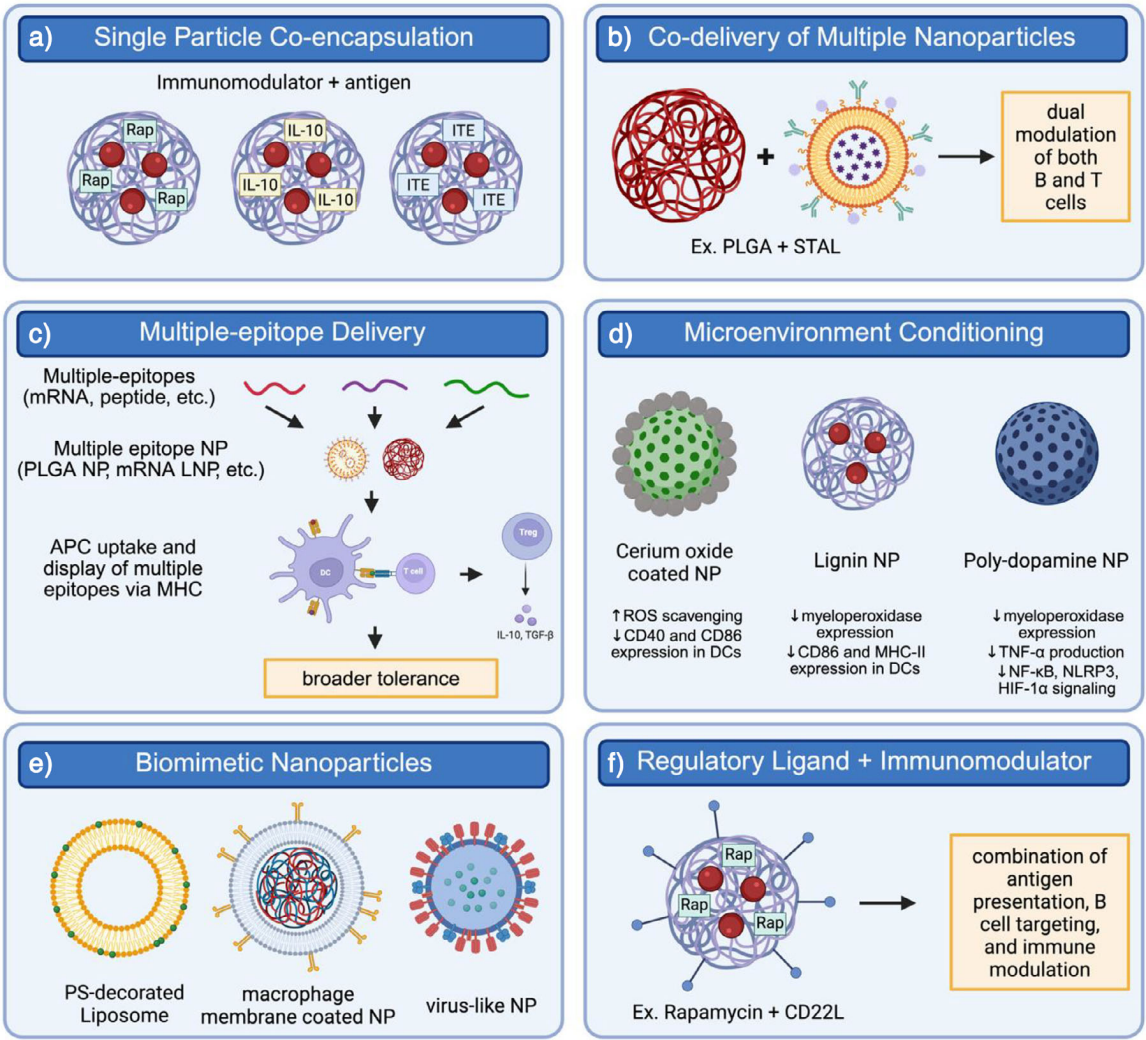
Nanoparticles can be engineered to simultaneously modulate multiple immune pathways. a) Single particle co‐encapsulation synchronizes antigen and immunomodulator delivery within the same APC. b) Co‐delivery of multiple nanoparticles (e.g., PLGA with STAL liposomes) enables parallel modulation of B and T cells. c) Multiple‐epitope delivery broadens tolerance by allowing APCs to present diverse epitopes. d) Microenvironment conditioning with redox‐active or anti‐inflammatory carriers (e.g., cerium oxide, lignin, polydopamine) reduces inflammatory signaling. e) Biomimetic nanoparticles mimic apoptotic or natural immune cues to drive tolerance. f) Regulatory ligand plus immunomodulator combinations (e.g., CD22L with rapamycin) integrate antigen presentation with direct immune suppression. Together, these approaches highlight how multifunctional nanoparticle design can achieve durable and antigen‐specific tolerance.

Co‐encapsulation of antigen and immunomodulator within a single nanoparticle enables coordinated uptake and intracellular processing by the same APC (Figure 3a). For example, Cappellano et al. encapsulated MOG35–55 peptide and recombinant IL‐10 into PLGA nanoparticles.^[^ ^40^ ^]^ In a murine EAE model, subcutaneous delivery of this platform significantly reduced disease severity by skewing DCs toward a tolerogenic phenotype and promoting Foxp3^+^ T_reg_ expansion. Similarly, Yeste et al. co‐delivered MOG antigen with the aryl hydrocarbon receptor complex (AhR) agonist ITE in DC‐targeting NPs, which suppressed EAE by driving DCs toward a tolerogenic phenotype favoring T_reg_ induction.^[^ ^95^ ^]^ In a mouse model of Graves’ disease, Chen et al. demonstrated that PLGA NPs loaded with the TSHR‐A antigen and rapamycin induced tolerance by targeting splenic DCs, inhibiting their maturation, and promoting T_reg_ proliferation.^[^ ^96^ ^]^ Beyond T cells, Brzezicka et al. demonstrated that hybrid NPs co‐loaded with CD22L ligand and rapamycin could simultaneously suppress autoreactive B and T cells in RA,^[^ ^45^ ^]^ highlighting how ligand–immunomodulator synergy enables dual‐arm tolerance (Figure 3f). In allergy, Liu et al. showed that co‐encapsulation of BLG peptide with CpG oligodeoxynucleotides in PLGA NPs reduced CD11b⁺ DCs in mesenteric lymph nodes, decreased BLG‐specific IgE, and increased IL‐10 and T_regs_ in a food allergy model.^[^ ^44^ ^]^ Notably, in this model, co‐encapsulated NPs more effectively dampened DC activation markers compared to co‐administration of single‐payload NPs. Collectively, these findings underscore that temporal and spatial coordination of antigen and immunomodulator cargo delivery is critical for reprogramming APCs and achieving antigen‐specific tolerance.

Complementary work with multi‐nanoparticle systems has reinforced this principle (Figure 3b). The previously discussed works by Srivastava et al. and Brzezicka et al. also highlighted the importance of multi‐nanoparticle delivery systems in autoimmune arthritis.^[^ ^45^, ^84^ ^]^ In these studies, combining STALs (B cell‐targeting CD22L liposomes) and PLGA‐R (T cell‐targeting rapamycin NPs) led to a significant reduction in autoantibodies and inflammatory cytokines in K/BxN mice. Notably, only the combination nanoparticle approach achieved full disease suppression, indicating that dual modulation of B and T cells with individual nanoparticle systems facilitates robust and durable tolerance. While co‐delivery of individual nanoparticles was effective, direct co‐encapsulation of antigens and immunomodulators into a single nanoparticle yielded stronger tolerogenic effects.

Multi‐valent and multi‐epitope nanoparticle formulations further enhance the breadth of immune reprogramming, particularly in poly‐antigenic conditions (Figure 3c). Pearson et al. showed that PLGA NPs encapsulating both PLP139–151 and PLP178–191 peptides led to superior EAE suppression and T_reg_ induction compared to single‐epitope particles.^[^ ^97^ ^]^ Similarly, Xu et al. demonstrated that liver‐targeted mRNA LNPs encoding a cocktail of Ara h2 epitopes provided greater suppression of peanut‐induced anaphylaxis than the most potent individual epitope.^[^ ^47^ ^]^ Collectively, these studies demonstrate that co‐encapsulation of cargos promotes antigen‐specific immune tolerance across diverse autoimmune and allergic conditions.

### 4.3 ROS‐Modulating and Antioxidant Tolerogenic Systems

Reactive oxygen species (ROS) are key amplifiers of inflammation in autoimmune diseases, driving DC maturation and pro‐inflammatory cytokine production. Reactive oxygen species (ROS) exert a dual role in autoimmune disease. Chronic ROS production contributes to tissue injury, antigen‐modification and inflammation in organ‐specific autoimmune settings. However, physiologic ROS are required for immune signaling, homeostasis and macrophage/DC function.^[^ ^98^, ^99^ ^]^ In many organ‐specific autoimmune diseases (for example, MS,^[^ ^100^ ^]^ T1D,^[^ ^101^ ^]^ and Hashimoto's Thyroiditis^[^ ^102^ ^]^) elevated ROS levels have been documented within phagocytes or parenchymal tissues at the site of inflammation, supporting the concept of local oxidative microenvironments rather than widespread lymphoid‐organ ROS up‐regulation.^[^ ^103^ ^]^ Accordingly, nanoparticle platforms designed to scavenge ROS may achieve the greatest impact when targeted to the specific diseased tissues or their draining lymph nodes, where oxidative stress is highest, rather than relying on systemic lymphoid‐organ targeting.

Nanoparticles can be engineered to scavenge ROS directly or respond to oxidative cues to modulate local immune response (Figure 3d). Nanoparticle‐based microenvironment conditioning synergizes with antigen delivery to promote tolerogenic APCs and T_reg_ expansion. There are several strategies to generate ROS‐reactive nanoparticles. First, Nguyen et al. enhanced the tolerogenic properties of mesoporous silica nanoparticles (MSNs) by decorating their surface with cerium oxide nanoparticles (CeNPs), which possess intrinsic ROS‐scavenging activity, using electrostatic interactions.^[^ ^55^ ^]^ In a murine EAE model, CeNP‐MSNs loaded with MOG peptide reduced CD86 and CD40 expression on CD11c^+^ DCs and F4/80^+^ macrophages and significantly increased splenic Foxp3^+^ T_regs_. Compared to MSNs without CeNPs, the hybrid NPs demonstrated superior disease suppression, indicating that antioxidative modulation enhances antigen‐specific tolerance. As previously discussed, in a different approach Phan et al. used lignin, a natural polyphenol with known antioxidant capacity, to coat the surface of LNPs encapsulating MOG antigen.^[^ ^90^ ^]^ In a murine model of EAE, lignin‐coated LNPs delivered antigen, suppressed oxidative stress in lymphoid tissues, and lowered expression of myeloperoxidase (MPO), a pro‐oxidant enzyme elevated in EAE. This tolerogenic platform also decreased DC expression of CD86 and MHC‐II and elevated Foxp3^+^ T cells, leading to prevention of disease onset. These results suggest a dual mechanism of immune reprogramming via ROS scavenging and antigen‐specific tolerance. Some biomaterials themselves are ROS‐responsive. For example, mesoporous polydopamine‐based (PDA) nanoparticles were used both as an intrinsic redox regulator and a MOG antigen carrier in a mouse model of EAE.^[^ ^52^ ^]^ This platform increased Foxp3^+^ T_regs_, reduced MPO levels, and fully prevented disease onset in vivo, even in late‐stage disease, underscoring their potent anti‐inflammatory and tolerogenic capacity.

In sum, these studies support the principle that tolerance induction is enhanced when antigen delivery is coupled with direct modulation of the inflammatory microenvironment. By buffering oxidative stress, ROS‐reactive nanoparticles not only suppress unwanted activation of innate immune cells but also favor the emergence of regulatory phenotype, representing a powerful tool for restoring immune balance in autoimmunity.

### 4.4 Biomimetic Nanoparticles

Biomimetic nanoparticles aim to replicate natural immunoregulatory processes, such as apoptotic cell clearance or immune cell signaling, to promote antigen‐specific tolerance (Figure 3e). These systems leverage the body's intrinsic recognition of “self” or “silent” signals to engage tolerogenic APCs without triggering inflammatory cascades.

Phosphatidylserine (PS)‐decorated liposomes mimic apoptotic cells, utilizing the efferocytosis pathway to induce semi‐mature, tolerogenic DCs. For example, Pujol‐Autonell et al. designed PS‐liposomes carrying diabetes autoantigen peptide that trafficked to the pancreas and pancreatic lymph nodes of non‐obese diabetic (NOD) mice, reducing insulitis and delaying T1D onset.^[^ ^104^ ^]^ Despite an increase in CD40 and CD86 expression, often associated with DC maturation, this platform increased expansion of both CD4^+^ Foxp3^+^ and Foxp3^−^ T cells, indicating functional tolerance. In a subsequent study, Pujol‐Autonell et al. developed PS‐liposomes encapsulating MOG_40–55_ peptide that induced tolerogenic DCs in vitro and reduced clinical disease severity in EAE mouse models.^[^ ^105^ ^]^ Interestingly, unloaded PS‐liposomes also modestly reduced disease scores, suggesting that PS itself contributes to baseline immunomodulation through apoptotic mimicry. Almenara‐Fuentes et al. further demonstrated PS‐liposome‐mediated antigen‐specific tolerance in mouse models of both RA (antigen‐induced arthritis; AIA) and myasthenia gravis (experimental autoimmune myasthenia gravis; EAMG).^[^ ^106^ ^]^ Rodríguez‐Fernández et al. applied PS‐liposomes to human disease.^[^ ^107^ ^]^ In DCs harvested from diabetic patients, PS‐liposomes downregulated co‐stimulatory markers and inflammatory cytokines, reinforcing the translational relevance of this approach. Finally, Tredicine et al. advanced the liposome platform with a dual‐layer architecture, an outer PS layer and an inner phosphatidic acid (PA) core, that better mimicked apoptotic bodies.^[^ ^108^ ^]^ In MS patient samples, this platform modulated macrophage and T cell phenotypes toward anti‐inflammatory profiles.

Beyond mimicry of apoptotic lipid signaling, nanoparticles can be engineered to mimic immune‐responsive cell surface ligands. For example, Zhong et al. engineered macrophage membrane‐coated nanodrugs that retained surface PD‐L1 and cytokine‐scavenging properties.^[^ ^109^ ^]^ In a collagen‐induced arthritis (CIA) mouse model, these biomimetic membrane‐coated NPs accumulated in inflamed joints and mediated immune suppression by enhancing local IL‐10 and TGF‐β production and inducing activation markers on DCs and B cells. Nanoparticles can also be engineered to mimic viruses, which are evolutionarily optimized for efficient nucleic‐acid delivery, by displaying epitopes recognized by the immune system. These virus‐mimetic particles exploit multivalent antigen presentation and intracellular cargo release to engage APCs in a context that promotes regulatory rather than effector responses. Furthermore, they offer additional advantages such as multivalent antigen display, efficient production via molecular‐farming, and scalable manufacturing as plant‐virus scaffolds. Zampieri et al. designed plant virus nanoparticles (pVNPs), based on cowpea mosaic virus (CPMV) and tomato bushy stunt virus (TBSV), that displayed autoimmune epitopes.^[^ ^110^ ^]^ In NOD mice, CPMV presenting p524opt peptide delayed diabetes onset through modulation of IL‐10 and IL‐2 skewing, although without significant T_reg_ expansion. In contrast, mice with RA treated with TBSV particles displaying pLIP1 or pFADK2 achieved full remission, increased Foxp3^+^ T_regs_, and suppressed inflammatory cytokines (TNF‐α, IL‐17, IFN‐γ). In both the NOD and CIA models, the plant‐virus nanoparticle served both as a peptide scaffold and an adjuvant, suggesting that one mechanistic advantage is the stabilization and high‐density multivalent display of auto‐antigen peptides on a viral capsid shell.

As demonstrated in the above studies, biomimetic nanoparticles mimic natural tolerogenic cues to reprogram immune responses without exogenous immunosuppression. The modularity and biocompatibility of biomimicry may be harnessed for the development of tolerogenic vaccines across autoimmune diseases.

### 4.5 Nucleic Acid‐Based Nanoparticle Delivery Platforms

Messenger RNA (mRNA) has emerged as a powerful tool for tolerogenic vaccine design. mRNA offers transient antigen expression without genomic integration risk and can be chemically modified to reduce innate immunogenicity. Unlike peptides, mRNA does not require chemical conjugation and can encode multiple epitopes. mRNA can be delivered to tissues or cells of interest with LNPs for targeted immune modulation. Indeed, mRNA‐LNPs have been used for a range of strategies to combat autoimmunity,^[^ ^49^ ^]^ such as ex vivo engineering of T cells with suppressive phenotypes or ex vivo engineering of B cell‐targeting chimeric antigen receptor (CAR) T cells.^[^ ^111^, ^112^ ^]^

Recent studies have demonstrated the promise of antigen‐encoding mRNA‐LNPs in tolerogenic vaccination. For example, Gomi et al. developed PS‐decorated LNPs encapsulating mRNA encoding the MOG_35–55_ antigen to promote immune tolerance in EAE.^[^ ^113^ ^]^ The PS shell mimicked apoptotic signals to engage tolerogenic dendritic cells, while the LNP core facilitated cytosolic mRNA release and antigen expression. In EAE mice, this platform reduced co‐stimulatory markers on splenic DCs and increased Foxp3^+^ T_regs_, delaying disease onset and clinical severity. As previously discussed, Xu et al. engineered mannose‐functionalized LNPs encapsulating mRNA encoding Ara h2 allergen epitopes to specifically target LSECs, which are known to induce T_reg_ responses.^[^ ^47^ ^]^ In mouse models of peanut anaphylaxis, both single and multi‐epitope formulations suppressed allergic reactions, serum IgE, and Th2 cytokines. Wang et al. designed spleen‐targeted mRNA‐LNPs encoding house dust mite antigens that selectively accumulated in splenic APCs, reducing eosinophilic infiltration in the spleen and increasing IL‐10 producing T_regs_. This platform suppressed allergic airway inflammation in a mouse model of asthma.^[^ ^48^ ^]^ Finally, Liu et al. extended tolerogenic mRNA delivery beyond autoantigen expression by designing LNPs encapsulating mRNA for PD‐L1. This platform reprogrammed APCs in vivo, reducing effector T cell activation and promoting regulatory phenotypes in a mouse model of lupus.^[^ ^114^ ^]^ Thus, co‐delivery of antigen‐encoding and checkpoint‐regulating mRNAs could offer a synergistic strategy for tolerogenic vaccines.

Taken together, mRNA‐LNPs enable the targeted delivery of autoantigens, allergen epitopes, or immunosuppressive proteins to induce spatially and temporally controlled immune tolerance, providing a modular strategy to design tolerogenic vaccines.

**Table 2:** Summary of recent approaches that apply nanomaterial selection, design, and delivery strategies for tolerogenic vaccines. Combinations of these techniques are discussed in greater detail in Section 3. Key: AIA, autoimmune arthritis; BMDC, bone marrow‐derived dendritic cell; cDC, conventional dendritic cell; DC, dendritic cell; EAE, experimental autoimmune encephalomyelitis; GD, graves’ disease; ITE, indole‐3‐thiol‐ethyl (AhR agonist); LE, lupus erythematosus; LSEC, liver sinusoidal endothelial cell; MBP, myelin basic protein; MCV, molluscum contagiosum virus; MG, myasthenia gravis; MOG, myelin oligodendrocyte glycoprotein; MS, multiple sclerosis; NP, nanoparticle; OVA, ovalbumin; PBMC, peripheral blood mononuclear cell; pDC, plasmacytoid dendritic cell; RA, rheumatoid arthritis; T1D, type 1 diabetes; T_regs_, regulatory T cells.

Biomaterial	Specific nanomedicine	Cargo	Target disease	Implicated immune pathways	Cell targets of interest
Metal‐based	Mesoporous silica NPs	MOG_35‐55_	MS^[^ ^55^ ^]^	MOG_35‐55_ antigen suppresses EAE development via Foxp3^+^ T_reg_ induction	CD11c^+^ DCs, F4/80^+^ macrophages, B cells, splenic CD4^+^ T cells
	Cerium oxide NPs	MOG_35‐55_	MS^[^ ^55^ ^]^	ROS‐scavenging shifts DC to tolerogenic phenotype	BMDCs, CD4^+^ T cells
	Zinc peroxide NPs	ZnO_2_, Catalase	RA^[^ ^93^ ^]^	Inactivation of OTUB1 deubiquitination to promote CCL5 degradation and induce tolerogenic DCs	DCs
	Lipid‐coated calcium phosphate NPs	Citrullinated peptides, rapamycin	RA^[^ ^115^ ^]^	Downregulation of DC activation markers (CD40, CD80, CD86)	DCs, T_regs_
	Gold NPs	MOG_35‐55_, ITE	MS^[^ ^95^ ^]^	AhR targeting to promote tolerogenic DCs	DCs
Polymer‐based	PLGA NPs	MOG_35‐55_, IL‐10, OVA, BLG peptide, rapamycin	MS, RA, AIA, GD, anaphylaxis^[^ ^40^, ^42^, ^45^, ^84^, ^96^, ^116^ ^]^	Inhibition of IFN‐γ and IL‐17 secretion, T cell anergy, Foxp3 expression upregulation	Spleen lymphocytes, PBMCs
	Nanogels	Mycophenolic acid	LE^[^ ^57^ ^]^	Downregulation of DC activation markers (CD40, CD80, CD86)	CD4^+^ T cells, BMDCs
Lipid‐based	LNPs	MOG_35‐55_ mRNA, peanut allergen epitope mRNA	EAE, anaphylaxis^[^ ^47^, ^113^ ^]^	Tolerogenic PS‐mediated signaling, inhibition of endosome maturation, T_reg_ expansion in spleen	T cells, M1/M2 macrophages, LSECs
	Liposomes	MBP, Human insulin, MCV, AChR146‐162, MOG_35‐55_ peptides	T1D, MS, MG, RA, MG ^[^ ^106^, ^107^, ^108^ ^]^	PS‐liposome induced IL‐10 secretion	B cells, cDCs, pDCs, macrophages, T_regs_
Protein‐based	Plant virus nanoparticles	p524 (T1D), pLIP1 (RA), pFADK2 (RA)	T1D, RA^[^ ^110^ ^]^	Liprin and focal adhesion kinase regulation	Foxp3^+^ T_regs_

## 5 Outlook for Nanoparticle‐Based Tolerogenic Vaccines

### 5.1 Current Scientific Challenges

There are several opportunities for improvement in tolerogenic vaccine design. First, autoantigens or allergens must be identified and characterized for each autoimmune or allergic disease. If the wrong antigen is selected, or if processing occurs in an immunogenic rather than tolerogenic context, the tolerogenic vaccine risks amplifying pathogenic responses rather than suppressing them. Several preclinical studies have demonstrated that antigen‐specific immunotherapies lacking appropriate design for tolerogenic presentation may inadvertently activate autoreactive T cells rather than suppress them.^[^ ^117^, ^118^ ^]^ To minimize this risk in nanoparticle‐based platforms, precise antigen/immunomodulator selection with known tolerogenic capacity is crucial.^[^ ^119^ ^]^ Further, the use of biomaterials or coatings with low innate immunogenicity and integration of regulatory cues (e.g., IL‐10/TGF‐β, apoptotic‐cell mimicry) can ensure aberrant immune activation is avoided. Second, the specificity of nanoparticle trafficking and biodistribution is currently limited. Even with targeting ligands or PEGylation, off‐target accumulation, especially in the liver, spleen, or lungs, can dilute therapeutic impact or provoke unintended immune effects. Even small modifications of surface chemistry can drastically alter nanoparticle fate, necessitating predictive and modular design frameworks.^[^ ^120^ ^]^ Finally, the durability and safety of tolerogenic vaccines requires further study. Prior to clinical translation, tolerogenic vaccines must reliably induce long‐lasting antigen‐specific tolerance without excessive systemic induction of the immune system.

### 5.2 Translational Challenges

Manufacturing of nanoparticle‐based tolerogenic vaccines, especially those incorporating multiple epitopes or ligands, pose significant cost and reproducibility challenges. Platforms that require long, multistep conjugations or surface modifications are typically performed in small batches, hindering their scalability. Stability of tolerogenic vaccines is another bottleneck, as surface‐modified nanoparticles may degrade or lose functional moieties after prolonged storage. Lipid and polymer sourcing, batch‐to‐batch consistency, and formulation stability must be optimized for industrial production and translation of multi‐functional tolerogenic vaccines. Next‐generation nanoparticles incorporating conjugation, combinatorial nucleic acid cargos, or biomimicry strategies will likely face a complex regulatory pathway involving material characterization and long‐term safety profiling, requiring strong partnership with regulatory bodies.^[^ ^121^ ^]^ Finally, vaccine hesitancy may impact patient acceptance for tolerogenic vaccines in the clinic, necessitating transparent communication about safety, reversibility, and antigen‐specificity of these platforms.

Despite these translational hurdles, early clinical trials using tolerogenic dendritic cells in MS (NCT03726307, 2024), peptide‐loaded dendritic cells in T1D (NCT04590872, 2024), and subcutaneous VLP‐based vaccines for peanut allergy have demonstrated antigen‐specific immune modulation without systemic immunosuppression.^[^ ^122^ ^]^ The liver‐targeted inverse vaccine ANK‐700 (Phase 1, NCT04602390) in relapsing‐remitting MS has completed enrollment and shown safety and biomarker trends for myelin‐reactive T cell suppression (NCT04602390, 2024). Moreover, the nanoparticle‐coupled epitope therapy TPM502 (Phase 2a, NCT05660109, 2024) in HLA‐DQ2.5 + celiac disease patients demonstrated a favorable safety profile, dose‐dependent reductions in IL‐2/IFN‐γ release by gluten‐specific T cells, and durable phenotypic shifts consistent with regulatory T‐cell induction. These studies provide proof‐of‐concept that precision immune tolerance is achievable in humans and motivate further development of nanoparticle‐based tolerogenic vaccines. However, not all antigen‐specific interventions have shown success. Several oral antigen‐therapy trials spanning autoimmune diseases failed to achieve clinical efficacy or, in some cases, raised safety concerns such as oral insulin trials in T1D (NCT00419562 and NCT01035801) and early peptide‐based trials in MS (NCT00079495). These failures underscore the importance of antigen selection, delivery context and immunomodulatory design in avoiding unwanted immune activation and ensuring durable tolerance.

## 6 Conclusion

Realizing the full potential of tolerogenic vaccines requires designing nanoparticle platforms that combine precise antigen delivery and context‐dependent immunomodulation. Biomaterial selection, nanoparticle physicochemical properties, and route of vaccine administration play a key role in the safety and efficacy of tolerogenic vaccines. To induce tolerance without systemic inflammation, nanoparticles can be engineered to engage multiple immune pathways simultaneously via conjugation, microenvironment conditioning, biomimicry, and cargo synchronization. These biomaterial innovations must be matched by scalable production, harmonized regulatory pathways, and thoughtful public communication for clinical translation. If these scientific and translational challenges are met, nanoparticle‐based tolerogenic vaccines have the potential to reshape how autoimmune and allergic diseases are treated, shifting from symptom management to long‐lasting reprogramming of the immune response.

## Conflict of Interests

The authors declare no conflict of interest.

## Data Availability

Data sharing is not applicable to this article as no new data were created or analyzed in this study.
